# Dynamic intermittent compression cryotherapy with intravenous nefopam results in faster pain recovery than static compression cryotherapy with oral nefopam: post-anterior cruciate ligament reconstruction

**DOI:** 10.1186/s40634-023-00639-3

**Published:** 2023-07-24

**Authors:** Mohamad K. Moussa, Nicolas Lefevre, Eugenie Valentin, Alain Meyer, Olivier Grimaud, Yoan Bohu, Antoinne Gerometta, Frederic Khiami, Alexandre Hardy

**Affiliations:** Department of Sports Surgery, Clinique du Sport, 75005 Paris, France

**Keywords:** Early recovery, ACL reconstruction, Morphine-free analgesia, Cryotherapy, Dynamic compression cryotherapy, PASS, Static cryotherapy

## Abstract

**Purpose:**

To evaluate the effectiveness of dynamic intermittent compression cryotherapy (DICC) (CryoNov®) with an intravenous nefopam-based pain management protocol (DCIVNPP) in reducing post-operative pain following anterior cruciate ligament reconstruction (ACLR) compared to static compression cryotherapy (SCC) (Igloo®) and oral Nefopam.

**Methods:**

This was a retrospective analysis of prospectively collected data including 676 patients who underwent primary ACLR in 2022. Patients were either in the DCIVNPP group or in the SCC (control group), and were matched for age, sex, and Lysholm and Tegner scores (338 per arm). The primary outcome was pain on the visual analogue scale (VAS), analyzed in relation to the minimal clinically important difference (MCID) and the Patient Acceptable Symptom State (PASS) thresholds for VAS. The secondary outcome was side effects.

**Results:**

Postoperative pain in the DCIVNPP group was less severe on the VAS than in the control group (*p* < 0.05). The maximum difference in the VAS between groups was 0.57, which is less than the MCID threshold for VAS. The DCIVNPP group crossed the PASS threshold for VAS on Day 3, sooner than the control group. The side effect profiles were similar in both groups except for higher rates of dizziness and malaise in the DCIVNPP group, and higher rates of abdominal pain in the control group. Most of the side effects decreased over time in both groups, with no significant side effects after Day 3.

**Conclusion:**

DCIVNPP effectively allows for faster pain recovery than in the control group. The difference in side effects between the protocols may be due to mode of administration of nefopam.

**Level of evidence:**

III.

## Introduction

Anterior Cruciate Ligament (ACL) is the most frequently reconstructed knee ligament [1]. ACL reconstruction (ACLR) procedure is known to be associated with moderate to severe pain, that can negatively affect knee function and extend hospital stays if it is not properly managed [2, 3], with an early postoperative Visual analogue scale (VAS) ranging from 2.5 to 5.4 depending on the pain protocol [4, 5]. Arthrogenic Muscle Inhibition (AMI) is a recently identified event following ACLR surgery. In AMI pain, inflammation, and changes in knee joint receptors lead to central inhibition of the quadriceps causing knee extension lag and affecting ACLR outcomes [6]. This shows the importance of obtaining optimal pain management. It is associated with high levels of patient satisfaction and is crucial for early hospital discharge, helping to avoid unnecessary hospitalization [2]. Furthermore, optimal pain management and ambulatory surgery can significantly decrease total healthcare costs following primary ACLR [7]. More than 77 randomized clinical trials have evaluated the best pain protocols including regional nerve blocks, intraarticular injections, intravenous and intramuscular injections, multimodal regimens, cryotherapy, and oral medications [8]. These studies have compared the effectiveness of these protocols on the VAS using different methods, including the evaluation of the minimally clinically important difference (MCID) and the Patient Acceptable Symptom State (PASS) [9–14]. The MCID is the smallest clinically significant improvement identified by patients, and is typically set between 1 and 1.4 for the VAS, depending on the study [10–12]. The PASS is the point when patients consider themselves to be well and satisfied with treatment [9, 13, 14]. The PASS for the VAS is typically set at 3.4 to 4 [9, 13–15].

Cryotherapy, a non-pharmacological treatment that reduces the local metabolism to alleviate pain and inflammation, has been found to be effective in reducing postoperative pain following ACLR [16–18]. There are different types of cryotherapies including non-compression cryotherapy (NCC), static compression cryotherapy (SCC), and dynamic intermittent compression cryotherapy (DICC). Although several RCTs and metanalyses have shown that compression cryotherapy is better than traditional forms of cryotherapy for reducing pain [18–20], there are only two studies comparing DICC and SCC in the literature [21, 22]. The first experimental study by Holwerda et al. compared tissue temperature changes and cardiovascular response between DICC with the GameReady® device to those of SCC using elastic ice wrapping [21]. This study showed that neither of these techniques caused acute cardiovascular strain and that there were no significant differences in the intramuscular temperature changes. The second preliminary study by Murgier et al., in 2014, including 39 patients in a case–control design, suggested that DICC could be more effective than SCC, with a 1.15 difference in VAS, although this was not statistically significant [22].

The aim of this study was to compare these two cryotherapy techniques by analyzing the effect of two morphine-free multimodal pain management protocols on early post-operative pain following ACLR. The first protocol used DICC with an intravenous-nefopam pain management protocol (DCIVNPP), which was compared to a similar protocol that used oral nefopam and SCC (control group). Both protocols were morphine-free, and nefopam was chosen due to its analgesic properties and its common usage in France. Nefopam is a non-opioid tricyclic drug developed in the late 1960s and early 1970s as an antidepressant that possesses analgesic properties [23]. Studies have not shown superiority of one route of administration over the other, but rather a difference in the potential for side effects [23–26].

We hypothesized that DCIVNPP would result in significantly lower pain levels according to the VAS, and that these patients would recover more rapidly with no increase in adverse effects such as nausea, dizziness, and malaise.

## Material and methods

### Study design

This is a retrospective analysis of prospectively collected data that included all patients who underwent primary ACL reconstruction in 2022 at a referral center for sports surgery.

Patients who underwent primary ACLR were included, while those who underwent revision surgery, and/or refused to participate in the study or to fill the online questionnaires were excluded.

Patients were divided into the DCIVNPP and the control groups, based on the change in pain management protocol that occurred mid-2022 at our institution. The control group included patients who received the traditional pain management protocol prior to the change, while the DCIVNPP group received the new protocol. The control group included patients matched to the DCIVNPP group for age, sex, Lysholm score [27], and Tegner score [28].

### Anesthesia, surgical procedures, and rehabilitation protocol

#### Surgery

Patients from both groups were operated on by 6 orthopedic surgeons specialized in sports surgery, using different ACLR techniques, mainly hamstring grafts (quadrupled semitendinosus, doubled gracilis and doubled semitendinosus, biofast technique [29] with or without lateral extraarticular procedure), and modified Macintosh procedures [30].

#### Anesthesia

Both groups received preoperative spinal anesthesia and an ultrasound-guided selective sensory nerve block of the saphenous nerve with ropivacaine in the adductor canal (20 mL ropivacaine 0.2%, equivalent of 40 mg [5, 31]). Both groups received peri-operative local ropivacaine injections (1 vial of ropivacaine 2%) at the incision site [5, 31].

#### Postoperative pain management

Both post-operative pain management protocols were detailed in Table 1.

**Table 1:** Cryotherapy and pain management protocols comparison between both groups

Aspect	Control Group	DCIVNPP Group
**Cryotherapy device**	Static compression cryotherapy with Igloo® device	Dynamic intermittent compression cryotherapy utilizing CryoNov® device from Orthonov
**Cryotherapy protocol**	Positioned by the surgeon at the end of the procedure and was turned on for 30 min and off for 2 h for 5 days. The device is removed at night	positioned by the surgeon at the end of the procedure, then programmed to turn on for 30 and 30 min off at low pressure for 5 days. The device is removed at night
**Common oral medication**	Classic 8-day systematic pain relief protocol. This protocol included 200 mg of oral Celecoxib twice daily, Lamaline (Paracetamol/opium) 500 mg/25 mg every 8 h, Omeprazol 20 mg/day, Paracetamol 500 mg every 8 h, and	
**Nefopam**	Oral Nefopam 20 mg/2 mL every 8 h	Intravenous administration of 3 vials of Nefopam 20 mg/2 ml diluted in 50 ml of normal saline solution (9%) a continuous IV for 12 h every 12 h,
**Other intravenous (IV) medication**^**a**^		1 vial of (IV) metoclopramide 10 mg/2 mL diluted in 50 mL of normal saline for 30 min every 12 h

*DCIVNPP* Dynamic intermittent compression cryotherapy (CryoNov®) with an intravenous nefopam-based pain management protocol

^a^This was done through hospitalization-at-home services, where a nurse would visit the patient every day to insert a peripheral IV line and deliver the medication

#### Rehabilitation protocol

The postoperative rehabilitation protocol was the same for all patients. The patient wore a hinged brace in full passive extension for several days and total weight bearing was allowed**.** The rehabilitation protocol began several days after surgery including closed chain isometric and eccentric quadriceps strengthening, and isometric hamstring co-contraction.

### Outcome measures

The primary outcome measure was the VAS on day 0, the first night, days 1, 2, and 3. The VAS results were compared between the groups and analyzed in relation to the MCID and PASS thresholds for the VAS [10–13].

Secondary outcomes included the rate of side effects such as nausea, malaise, dizziness, anxiety, and stomach pain.

The MCID threshold for VAS, typically ranging from 1 to 1.4 [10–13], and the PASS thresholds for the VAS, typically ranging from 3.2 to 4 [13, 15], were derived from relevant literature. In our analysis, we opted to utilize the highest reported values for MCID and lowest for PASS to facilitate a more conservative comparison between the groups as we did not find validation for these values in French population.

### Collected data

The following information was collected: age, sex, body mass index (BMI), preoperative IKDC subjective score, Lysholm score [27], Tegner score [28], type of sport, type of ACLR, addition of anterolateral reconstruction to the procedure, presence of meniscal lesions, type of hospitalization, and delay to surgery. Data for this study was collected prospectively by the French Prospective Anterior Cruciate Ligament Reconstruction Cohort Study (FAST, ClinicalTrials.gov Identifier: NCT02511158) and was compiled using Websurvey® software. Surgeons completed entries for the medical history, physical examination findings, work-up, and follow-up, while patients completed the questionnaires and scores.

### Ethical consideration

The study received approval from the center’s Ethics Committee (CPP-IDF-VI). All participating patients provided informed consent when they filled out the online survey.

### Study size

**Fig. 1:**
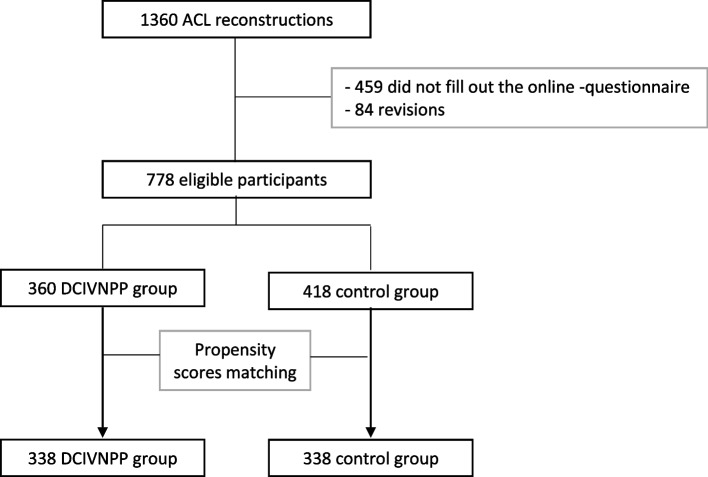
Flow chart of the study design

A total of 1360 ACLR procedures were performed at our institution in 2022. Five hundred and forty-three of these were excluded from the study, 459 for failure to fill out the online questionnaire and 84 were revision ACLR**.** Thus, a total of 778 patients were eligible for the study, 360 in the DCIVNPP group and 418 in the control group (Fig. 1).

The power calculation was conducted based on the hypothesis that the new protocol would lead to a minimum 10% reduction in pain compared to a previous study by the same institution, with a post-operative VAS score of 5 [32]. Using the Mann–Whitney method, the sample size was estimated to be 668 patients (334 per arm) to detect a significant difference of approximately 0.5 (assuming a standard deviation of 2.4). This sample size was determined to achieve a statistical power of 80% and a type I error rate of 5%.

Selection bias was minimized in both treatment groups by matching the study population using a propensity score. Participants were matched in a 1:1 ratio using a logit scale with a caliper width equal to 0.2. The matched variables included age, gender as well as Lysholm score [27], Tegner score [28] before surgery.

### Statistical analysis

Categorical variables were presented as percentages while continuous variables were presented as means and standard deviations. Comparisons were performed with the Fisher exact test or the Chi square test for categorical data and the Student t-test or Mann Whitney test for continuous variables, when appropriate.

A *p* value < 0.05 was considered to be statistically significant. All statistical analyses were performed using R software (version 4.2).

## Results

### Patient characteristics (Table 2)

**Table 2:** Patient characteristics before surgery

	**DCIVNPP group**	**Control group**	***P*** **-value**
	*N* = 338	*N* = 338	
**Age, mean (sd)**	30.1 (9.9)	30.3 (10.8)	0.80
**Gender, N (%)**			0.75
Male	197 (58%)	202 (60%)	
Female	141 (42%)	136 (40%)	
**BMI, mean (sd)**	24.4 (4.6)	24.2 (4.5)	0.27
**Side of the injury, N (%)**			0.62
Right	182 (54%)	174 (52%)	
Left	155 (46%)	162 (48%)	
Missing data	1	2	
**Hospitalization, N(%)**			0.09
Ambulatories	292 (86%)	274 (81%)	
Classique	44 (14%)	60 (19%)	
Missing data	2	4	
**Subjective IKDC score before surgery, mean (sd)**	59.7 (15.7)	58.6 (17.2)	0.49
**LYSHOLM score before surgery, mean (sd)**	72.5 (16.3)	72.3 (17.6)	0.97
**TEGNER score before surgery, mean (sd)**	6.9 (2.0)	7.0 (2.0)	0.72
**Sport levels, N(%)**			0.84
Competition	129 (38%)	132 (39%)	
Occasional leisure	38 (11%)	34 (10%)	
Regular leisure	153 (45%)	150 (44%)	
Professional	9 (3%)	14 (4%)	
Sedentarys	9 (3%)	8 (2%)	
**Type of sports, N (%)**			0.44
No sport	8 (2%)	6 (2%)	
Pivot contact (football, hand, rugby, basket, judo)	175 (52%)	196 (58%)	
Pivot without contact (tennis, ski, badminton, volley)	103 (31%)	83 (25%)	
Without pivot (jogging, bicycle. swimming)	52 (15%)	53 (16%)	
**Delay injury—surgery in month, mean (sd)**	4.0 (3.9)	4.3 (5.9)	0.41
Missing data	9	3	
**Type of grafts used for anterior cruciate ligament reconstruction**			< 0.0001
Simple Doubled semitendinous, doubled gracilis	134 (40%)	163 (49%)	
Biofast doubled semitendinous, doubled gracilis	34 (10%)	13 (4%)	
Quadrupled semitendinous techniques	143 (42%)	55 (17%)	
Modified Macintoch Fascia lata technique	14 (4%)	94 (28%)	
Bone patellar tendon bone technique	12 (4%)	8 (2%)	
Missing data	5	1	
**Associated of lateral stabilizing surgery such as Lemaire tenodesis or anterolateral ligament reconstruction**			
Yes	135 (40%)	71 (21%)	< 0.0001
No	202 (60%)	266 (79%)	
Missing data	1	1	
**Association with meniscal lesion**			0.12
Yes	151 (45%)	130 (39%)	
No	184 (55%)	204 (61%)	
Missing data	3	4	

*DCIVNPP* Dynamic intermittent compression cryotherapy with intravenous-nefopam pain management protocol, *SD* standard deviation

Both groups were statistically comparable. Patients’ mean age was 30.1 ± 9.9 in the DCIVNPP group and 30.3 ± 10.8 in the control group (*p* = 0.80) and there were more males in both groups (58% for DCIVNPP, 60% for control). The BMI (24.4 vs 24.2), the reoperative subjective IKDC, the Lyshlom and Tegner scores were also comparable between the groups (59.7/58.6), (72.5/72.3), and (6.9/7.0) respectively. There were more outpatient surgeries in the DCIVNPP group (292/338, 86%) than in the control group (274/338, 81%) (*p* = 0.09). The level and type of sports were comparable. There was no difference in time to surgery between both groups.

The doubled semitendinous doubled gracilis (134, 40%) and the quadrupled semi-tendinous (143, 42%) were the most frequent grafts in the DCIVNPP group while the doubled semitendinous, doubled gracilis (163, 49%) and the Macintoch Fascia lata technique (94, 28%) (*p* < 0.0001) were the most frequent in the control group. Lateral stabilizing surgery was performed in 40% of the DCIVNPP group (135 patients) vs 21% (71 patients) in control group (*p* < 0.0001). An associated meniscal lesion was not significantly different between the DCIVNPP and control group: 45% (151 patients) and 39% (130), respectively (*p* = 0. 12).

### Post-operative VAS (Tables 3 and 4)

**Table 3:** Comparison of the pain between the two groups

	**DCIVNPP group**	**Control group**	**Mean difference**	**IC (95%)**	***P*** **-value**
**VAS score Day 0, mean (sd)**	5.36 (2.7)	5.93 (2.7)	0.57	[0.16—0.98]	0.004
**VAS score first night, mean (sd)**	5.11 (2.4)	5.63 (2.4)	0.52	[0.16—0.88]	0.002
**VAS score Day 1, mean (sd)**	5.40 (2.4)	5.93 (2.6)	0.53	[0.15—0.91]	0.002
**VAS score Day 2, mean (sd)**	4.31 (2.3)	4.84 (2.4)	0.53	[0.18—0.88]	0.005
**VAS score Day 3, mean (sd)**	3.54 (2.3)	4.10 (2.3)	0.56	[0.21—0.91]	0.001

*PASS* patient acceptable symptoms state, *VAS* visual analogue scale, *DCIVNPP* Dynamic intermittent compression cryotherapy with intravenous-nefopam pain management protocol, *SD* standard deviation

**Table 4:** Percentage of patients crossing the patient acceptable symptoms state (PASS) threshold for pain measured by the visual analogue scale

	**Percentage of patients crossing the PASS for VAS**		***p*** **-value**
	**Control group**	**DCIVNPP group**	
**Day 0**	97 (28.7%)	128 (37.9%)	0.01
**First night**	94 (27.8%)	122 (36.1%)	0.03
**Day 1**	99 (29.3%)	144 (42.6%)	< 0.01
**Day 2**	147 (43.5%)	185 (54.7%)	< 0.01
**Day 3**	190 (56.2%)	237 (70.1%)	< 0.01

*PASS* patient acceptable symptoms state, *VAS* visual analogue scale, *DCIVNPP* Dynamic intermittent compression cryotherapy with intravenous-nefopam pain management protocol

Patients in the DCIVNPP group had statistically significant lower levels of post-operative pain on the VAS on Day 0, the first night and Days 1, 2, and 3 compared to the control group (*p* = 0.004, 0.002, 0.002, 0.005, and 0.001 respectively). Mean VAS scores for the DCIVNPP group were 5.36 (SD 2.7), 5.11 (SD 2.4), 5.40 (SD 2.4), 4.31 (SD 2.3) and 3.54 (SD 2.3) respectively, while the mean scores for the control group were 5.93 (SD 2.7), 5.63 (SD 2.4), 5.93 (SD 2.6), 4.84 (SD 2.4) and 4.10 (SD 2.3), respectively (*p* < 0.05) (Table 2).

The maximum difference in the VAS between the groups was 0.57 on day 0 [0.16—0.98] and 0.56 [0.21—0.91] on day 3. The DCIVNPP group consistently demonstrated higher percentages of patients crossing the PASS threshold for pain (< 0.05). On day 3, a significantly greater proportion of patients in the DCIVNPP group (70.1%) crossed the PASS threshold compared to the control group (56.2%) (Table 4).

### Side effects (Table 5)

**Table 5:** Side effects after surgery

	**DCIVNPP group**	**Control group**	***P*** **-value**
**Just after surgery—Day 0**			
**Side effects, N (%)**			
None	196 (61%)	217 (68%)	0.12
Nausea	40 (12%)	49 (15%)	0.36
Malaise	38 (12%)	30 (9%)	0.31
Dizziness	78 (24%)	54 (17%)	0.02
Anxiety	21 (7%)	16 (5%)	0.41
Abdominal pain	5 (2%)	9 (3%)	0.42
Missing data	16	13	
**Getting up, N (%)**			0.09
Yes	177 (52%)	151 (45%)	
No	161 (48%)	187 (55%)	
**First night after surgery**			
**Side effects, N (%)**			
None	237 (72%)	245 (74%)	0.60
Nausea	22 (7%)	28 (8%)	0.76
Malaise	17 (5%)	17 (5%)	1
Dizziness	54 (15%)	33 (9%)	0.04
Anxiety	23 (6%)	21 (6%)	0.76
Abdominal pain	4 (1%)	15 (5%)	0.02
Missing data	9	7	
**First day after surgery—Day 1**			
**Side effects, N (%)**			
None	223 (68%)	227 (68%)	0.99
Nausea	30 (9%)	40 (12%)	0.26
Malaise	22 (7%)	28 (8%)	0.46
Dizziness	53 (16%)	44 (13%)	0.32
Anxiety	17 (5%)	14 (4%)	0.58
Abdominal pain	7 (2%)	18 (5%)	0.04
Missing data	11	6	
**Getting up, N (%)**			0.09
Yes	285 (84%)	271 (80%)	0.32
No	53 (16%)	67 (20%)	
**Second day after surgery—Day 2**			
**Side effects, N (%)**			
None	252 (76%)	258 (77%)	0.85
Nausea	29 (9%)	30 (9%)	1
Malaise	12 (4%)	11 (3%)	0.84
Dizziness	30 (9%)	23 (7%)	0.32
Anxiety	11 (3%)	10 (3%)	0.83
Abdominal pain	10 (3%)	16 (5%)	0.32
Missing data	6	3	
**Getting up, N (%)**			0.92
Yes	309 (91%)	305 (90%)	0.70
No	29 (9%)	33 (10%)	
**Third day after surgery—Day 3**			
**Side effects, N (%)**			
None	259 (78%)	280 (84%)	0.06
Nausea	18 (5%)	19 (6%)	1
Malaise	10 (3%)	2 (1%)	0.02
Dizziness	21 (6%)	12 (4%)	0.11
Anxiety	7 (2%)	10 (3%)	0.63
Abdominal pain	12 (4%)	14 (4%)	0.99
Missing data	4	3	
**Getting up, N (%)**			0.92
Yes	325 (92%)	318 (92%)	0.26
No	13 (4%)	20 (6%)	

*DCIVNPP* Dynamic intermittent compression cryotherapy with intravenous-nefopam pain management protocol

Overall, the side effect profile of the DCIVNPP treatment were comparable to that of the control group, with some exceptions: the DCIVNPP group had higher rates of dizziness on day 0 and the first night (78 [24%] and 54 [15%], respectively) compared to 54 (17%) and 33 (9%) for the control group. They had also a higher rate of malaise on the third day (7 [3%]) compared to 18 (1%) for the control group. However, the control group had higher rate of abdominal pain on the first night and day 1 (4 [1%], and 7 [2%] respectively) compared to 15 (5%) and 18 (5%) respectively for the DCIVNPP group. Furthermore, the incidence of side effects decreased over time in both the DCIVNPP and control groups, from 39 and 32%, respectively, on Day 0 (*p*-value = 0.12), to 22% and 16%, respectively, on Day 3 (*p*-value = 0.06).

## Discussion

This cohort study evaluated postoperative pain management and the secondary side effects of DCIVNPP following ACLR. The main finding of this study is that DCIVNPP led to a faster pain recovery than the control group.

Our study consistently showed that a higher percentage of patients in the DCIVNPP group crossed the threshold for PASS at each day post-operative assessment. Specifically, by day 3, more than 70% of patients in the DCIVNPP group reached the acceptable symptom state. These findings suggest that the DCIVNPP protocol is effective in facilitating a faster recovery and achieving satisfactory symptom control in the early post-operative period. This faster recovery may have important clinical implications because it suggests that the DCIVNPP protocol may be more effective in reducing pain and improving patient satisfaction in the early post-operative period which can influence patient recovery, rehabilitation, and overall satisfaction [2]. The use of PASS in our assessment is important because the results in the literature suggest that PASS can serve as a benchmark to evaluate treatment efficacy [23, 33]. Recent research has shown that PASS could be used to accurately predict the level of sports activity that can be resumed after shoulder surgery [23, 33].

On the other hand, the clinical significance of a 0.57 decrease in VAS, found in our study, is limited because it does not meet the MCID threshold for this score, which is typically set at a decrease of between 1 and 1.4 points [10–13]. Nevertheless, although the statistically significant difference may not be clinically significant, it still indicates a reduction in pain. This modest reduction in pain is likely responsible for the observed faster recovery in the DCIVNPP group.

The early postoperative VAS scores in our study (3.54 to 5.4) are slightly higher than those reported in the literature, which typically range from 2.5 to 5.4 [4, 5, 32]. This is probably because there was no morphine or any step 3 analgesics for pain management in our protocol, and that our VAS data was collected online, potentially allowing for less external influence on the patient's reported pain levels.

In the current study, we hypothesize that the most important element to the DCIVNPP is the DICC component. There are very few studies in literature comparing DICC to SCC. This include one experimental study by Holwerda et al. [21] and one therapeutic study by Murgier et al. [22]. In 2014, Murgier et al. performed a preliminary study in 39 patients, comparing DICC (using GameReady®) to SCC (using IceBand®). They concluded that DICC decreases the need for analgesic drugs following ACLR and improves postoperative recovery of knee range of motion [22]. However, the superiority of DICC group in their study was only based on lower VAS scores, and the *p*-values were not statistically significant [22]. Our study, however, shows a significant optimization of pain management, thus providing more solid evidence for the effectiveness of the DICC in reducing postoperative pain, resulting in faster recovery. Holwerda et al. performed an experimental study that compared the changes in tissue temperature (on the skin surface and in the quadriceps muscle) and cardiovascular response (mean arterial pressure, heart rate, and forearm blood flow) between the DICC using the GameReady® device and the SCC using elastic ice wrapping [21]. The study found no acute pathological cardiovascular strain with either technique, with a physiological response of a 5-min increase in mean arterial pressure (reaching a maximum of 6 mmHg). Also, there was no significant intramuscular temperature difference between the two modalities. SCC resulted in a temperature change of -14 ± 2 °C, while DICC was associated with a change of -7 ± 3 °C to -11 ± 6 °C, depending on the programmed compression pressure. This study provides further insight into the safety profile and the underlying changes associated with the DICC (GameReady®) [21]. In 2016, Song et al. performed a meta-analysis to compare the effects of compressive cryotherapy with NCC alone and found that compressive cryotherapy resulted in significantly better outcomes [20]. Similar results were reported in more recent studies [19, 34]. The metanalysis by Davey et al. in 2021 only identified 11 RCTs studying the effect of compression cryotherapy following ALCR, and none of them compared the different types of compression [35].

Our results show that the adverse effect profile of DCIVNPP treatment was comparable to that of the control group, with slightly higher rates of malaise and dizziness and lower rates of abdominal pain. We hypothesize that these changes are attributed to the mode of administration of Nefopam, which may influence these types of nonspecific adverse effects. Although the exact mechanism of action of Nefopam is unclear, it is thought to act centrally by inhibiting the reuptake of serotonin, norepinephrine, and dopamine [23]. In addition, nefopam may have some anti-inflammatory effect [25]. By means of these mechanisms of action, nefopam possess analgesic properties [36].

Although there is very little literature comparing the oral and intravenous forms of Nefopam, certain authors have suggested that there may be decreased tolerance associated with intravenous administration [24, 26]. Common adverse effects of Nefopam include dry mouth, drowsiness, dizziness, headache, nausea, and constipation. These adverse effects are more likely to occur with high dosages or prolonged use. More severe adverse effects of Nefopam, such as flushing, hypertension, tachycardia, and even seizures, may occur with rapid administration [24, 26]. However, none of these severe adverse effects were reported in our study.

The main limitation of the study is that it is non-randomized, which may create a selection bias. To control this potential bias, we used a matching strategy based on propensity scores. Lack of randomization can also create performance bias. The DCIVNPP group receives more attention from a nurse, which may make them feel more comfortable and have better outcomes, leading to an overestimation of the effectiveness of the tested protocol. Additionally, the surgical technique varied among patients, introducing potential bias into the study. For example, lateral extraarticular tenodesis was less frequently performed in the control group, which could potentially result in either reduced pain due to a lesser extent of the procedure, or conversely, increased pain as a consequence of potential residual instability. Another limitation of this study is the absence of specific validation studies for the MICD and PASS values in the French population. However, we addressed this limitation by utilizing a range of values from multiple references and selecting the highest reported MCID threshold and lowest reported PASS threshold for our analysis. Finally, a detection bias may have occurred as patients in the DCIVNPP group may have reported less pain due to their belief that they are receiving a more advanced treatment.

These findings should be extrapolated with caution. Further studies are needed, in particular a randomized controlled trial comparing the safety and effectiveness of this protocol to other cryotherapy and pain management techniques. Moreover, more studies are needed to determine if the 1-day-faster early recovery can be extended to longer-term faster recovery, such as returning to play after ACLR.

## Conclusion

In conclusion, this study provides preliminary evidence for the effectiveness of DICC with IV nefopam in reducing early post-operative pain following ACLR. The main finding of this study is that patients in the DCIVNPP group cross the PASS threshold for pain faster. The route of nefopam administration may be associated with different side effect profiles: intravenous nefopam may increase the risk of dizziness and malaise while oral nefopam may increase the risk of abdominal pain.
